# Tibial cutting guide (resector) holding pins position and subsequent risks of periprosthetic fracture in unicompartmental knee arthroplasty: a finite element analysis study

**DOI:** 10.1186/s13018-021-02308-6

**Published:** 2021-03-22

**Authors:** Elvis Chun-sing Chui, Lawrence Chun-man Lau, Carson Ka-bon Kwok, Jonathan Patrick Ng, Yuk-wah Hung, Patrick Shu-hang Yung, Jason Chi-ho Fan

**Affiliations:** 1 Department of Orthopaedics and Traumatology, Faculty of Medicine, The Chinese University of Hong Kong, The Prince of Wales Hospital, Room 74029, 5/F, Lui Che Woo Clinical Science Building, Shatin, Hong Kong SAR; 2 Department of Orthopaedics and Traumatology, Alice Ho Miu Ling Nethersole Hospital, Taipo, Hong Kong

**Keywords:** Biomechanics, Unicompartmental knee arthroplasty, Finite element analysis, Periprosthetic fracture

## Abstract

**Background:**

Periprosthetic fracture of the tibia after unicompartmental knee arthroplasty has been reported to be associated with excessive pin holes created for stabilization of the cutting guide. However, fractures have also been reported in cases using two pins as in the method suggested by the manufacturer. It is currently unclear whether variations in pinhole positions make a difference in proximal tibial fracture risk.

**Methods:**

Finite element models were constructed using Chinese female bone computed tomography images, with bone cuts made according to the surgical steps of implanting a fixed bearing unicompartmental arthroplasty. Four combinations of pinholes (pins placed more closely to the medial tibial cortex or centrally along the mechanical axis as allowed by the tibial cutting guide) created for tibial cutting guide placement were tested by finite element analyses. Testing loads were applied for simulating standing postures. The maximum von Mises stress on the tibial plateau was evaluated.

**Results:**

Pinhole placed close to the medial edge of the proximal tibial plateau is associated with the highest stress (27.67 Mpa) and is more likely to result in medial tibial fracture. On the contrary, pinhole placed along the central axis near the tibial tuberosity has the lowest stress (1.71 Mpa) and reflects lower risk of fracture.

**Conclusion:**

The present study revealed that placing tibial cutting guide holding pins centrally would lower the risks of periprosthetic fracture of the medial tibial plateau by analyzing the associated stress in various pin hole positions using finite element analysis.

## Introduction

Unicompartmental knee arthroplasty (UKA) in appropriately selected patients is one of the successful treatment options for unicompartmental osteoarthritis with 10-year survivorship that ranges from 91.4 to 98% [1, 2]. UKA offers several potential advantages over total knee arthroplasty (TKA) including less-invasive surgical exposure, preservation of native bone stock, retention of the cruciate ligaments, lower perioperative morbidity and enhanced postoperative recovery [3]. However, when failures do occur, they can be challenging to manage. Periprosthetic fracture after UKA, although rare, is one of the possible complications and may necessitate complex revision and restriction in patient activities [4]. It commonly presents as medial tibial plateau stress fracture underneath the UKA tibial baseplate. As it progresses, loosening of tibial baseplate stability, tibial plateau collapse and progressive varus deformity would result.

It is important to identify risk factors to avoid periprosthetic fracture if possible. A previous study has reported coronal alignment of more than 6° varus or any valgus of a tibial component substantially increases fracture risks in the medial compartment [4]. Low bone mineral density (BMD) in the proximal tibia and drilling of multiple pinholes at the proximal tibial during tibial cutting guide placement are also suggested to increase the risk of fracture [5, 6]. Although drilling of multiple pinholes is not recommended, two holding pins for stabilizing the tibial cutting guide onto the tibia are an integral part of the design in various brands of UKA. However, various publications have reported occurrence of fractures using only two holding pins following the surgical technique instructions of the UKAs’ manufacturers [7–9]. It is unknown in current literature how different positions of these holding pins and hence the resultant pinholes would affect the subsequent risks of tibial fracture.

Finite element analysis (FEA) has been employed by engineers to determine how devices or structures may behave under different circumstances, and it is increasingly being used in the orthopaedics field to simulate the behavior of orthopaedic implants under different situations [10–13]. With more powerful and advanced computer systems available, orthopaedic implants are increasingly assessed by FEA to understand their mode of failure and clinical limitations, in order to improve clinical safety.

The purpose of the present study was to use FEA to analyze the risks of periprosthetic fracture with different tibial guide holding pin positions.

## Materials and methods

### Development of the three-dimensional model

**Fig. 1:**
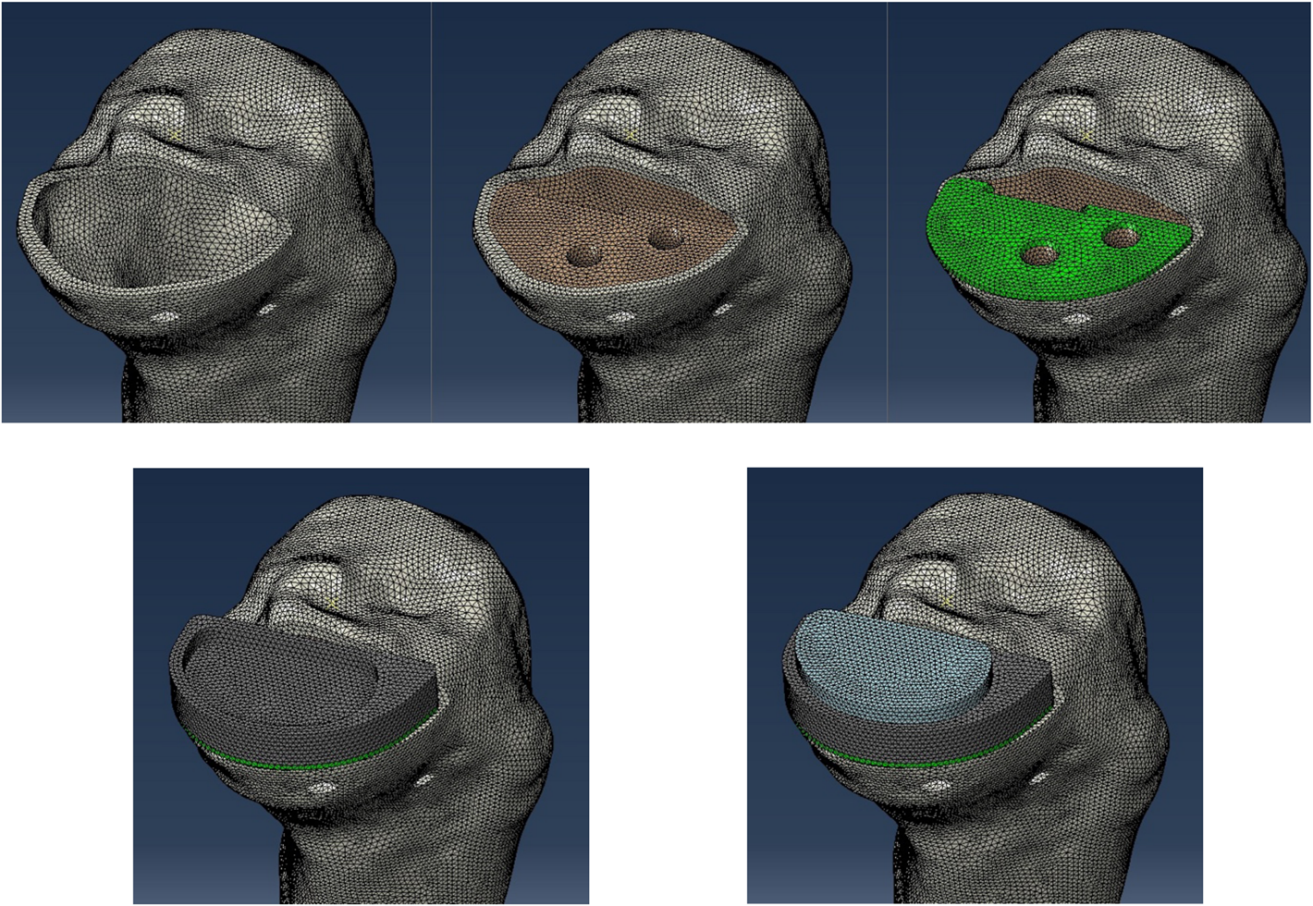
Separate meshes of the cortical bone, trabecular bone, the cement layer, tibial prosthesis tray and the polyethylene insert

The reconstructed continuum-based tibial models from the computerized tomographic image (Light Speed VCT; GE Medical System, General Electric Company, USA; slice thickness: 1.25 mm; image resolution: 512 × 512 pixels) of a Chinese female tibial bone were used in the present study, with reference to comparable study of FEA on lower limb [12, 14–20]. The medical records and radiological images for the subject showed neutral lower limb alignment without any anatomical abnormality, or previous operation. Digital CT data of the tibia was imported and segmented using the 3D model reconstruction software Mimics (version 14.0; Materialise, Belgium), which was used to generate a 3D geometrical surface of the tibia at full extension. The cortical structure and trabecular structure were separated as well (Fig. 1).

### Alignment definition

Knee center was defined by the center of posterior condylar rotational axis while the ankle center was defined by the center of the best fit cylinder matching the articular surface in the distal tibia. The sagittal shaft axis of the tibia was defined by the line connecting the knee center and the ankle center.

### Bone cut, model creation and prosthesis assembling

**Fig. 2:**
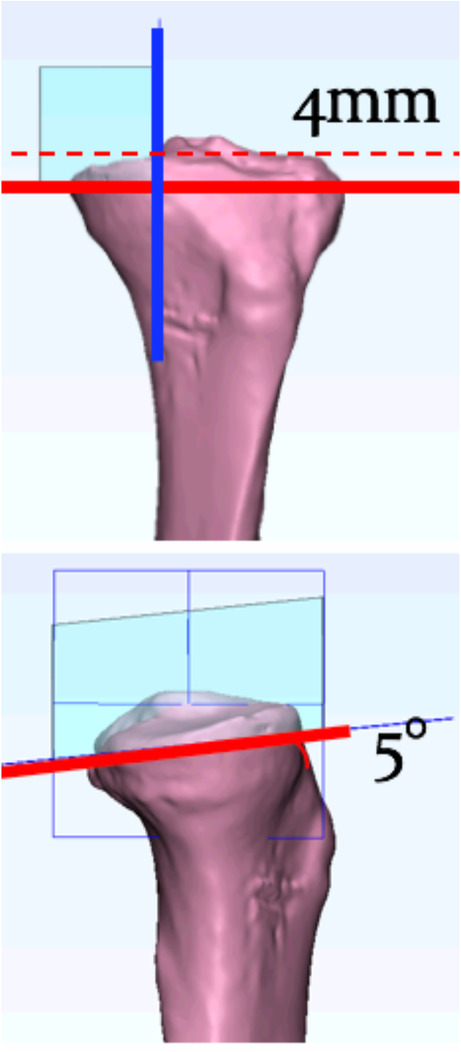
Transverse cut of the proximal tibia with a depth of 4 mm below the medial tibial plateau, with a posterior slope of 5° to the tibial shaft axis, and perpendicular to the coronal tibial axis

To simulate the technique of bone cut and prosthesis assembly, they were guided by a clinician. The virtual bone cut was performed to implant a medial unicompartmental knee arthroplasty referring to the instruction on the surgical technique manual of the Zimmer Unicompartmental High Flex Knee system, ZUK (Zimmer Inc., Warsaw, IN, USA) on the computer tomography model of the tibia of a Southern Chinese. A transverse cut of the proximal tibia was made to a depth of 4 mm below the medial tibial plateau, with a posterior slope of 5° to the tibial shaft axis, and perpendicular to coronal tibial axis (Fig. 2). The sagittal cutting plane was defined by the plane perpendicular to the transverse cutting plane and passing through the medial edge of the medial tibial spine.

**Fig. 3:**
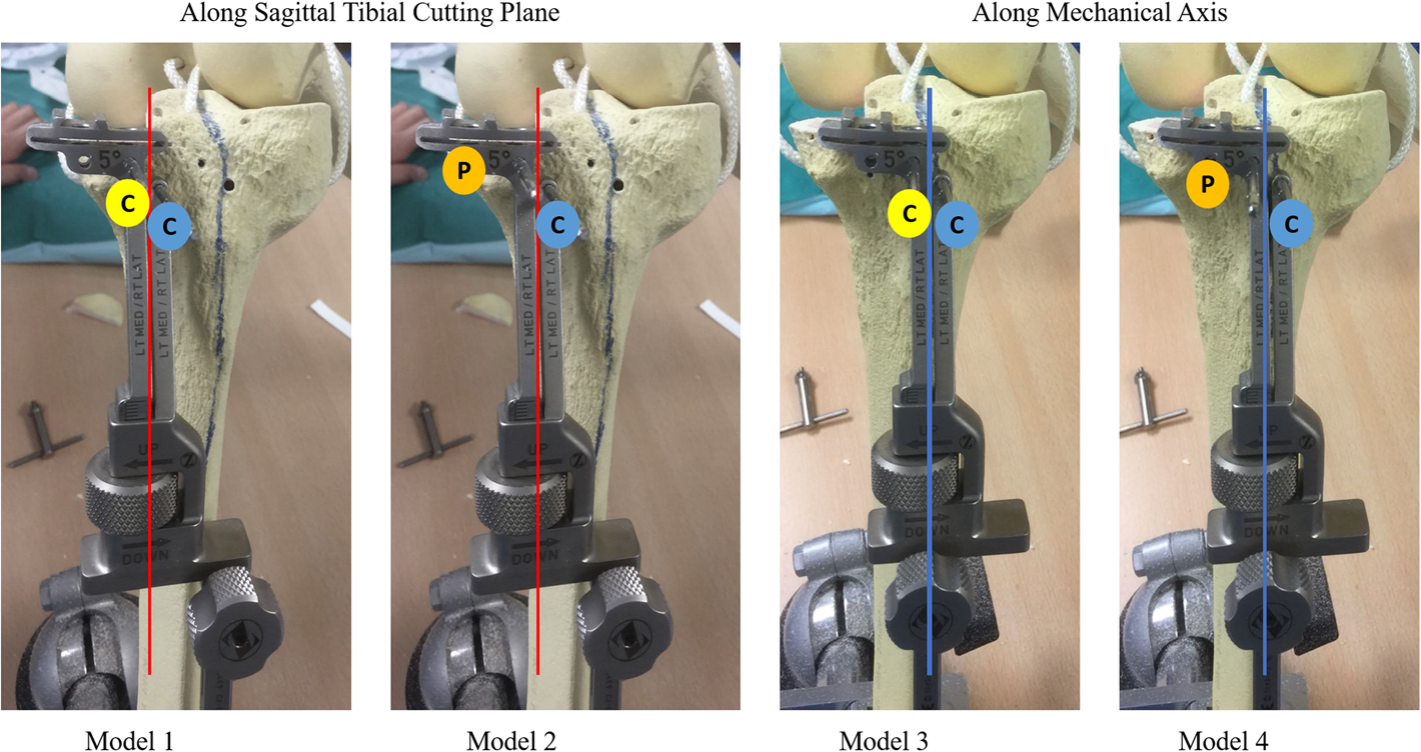
Four pin configuration models

Two pin holes were placed according to the design of the tibial cutting guide. Four pin configuration models were produced from using two combinations of pin holes and horizontal translation of cutting platform to give the following configuration (Fig. 3):

- Model 1: Two central pins, both along sagittal tibial cutting plane
- Model 2: One central pin along sagittal tibial cutting plane and one peripheral pin
- Model 3: Two central pins, both along tibia mechanical axis
- Model 4: One central pin along tibia mechanical axis and one peripheral pin

Remarks: In models 1 and 2, the tibial guide is aligned with the sagittal cutting plane. Models 3 and 4 are established by a parallel horizontal shift of the proximal part of the cutting guide laterally to align with the tibia mechanical axis (i.e. shifting respective models 1 and 2 laterally using the M/L slide adjustment at the midshaft of the assembly to re-position), while maintaining the transverse cut perpendicular to mechanical axis.

The medial unicompartmental knee prosthesis was redesigned using reverse engineering method by Solidworks software (Dassault Systèmes, USA). It was assembled on the medial tibia according to surgery requirements as stated on the surgical technique manual of the ZUK. The cement fixation was modeled by interposing a 2.0-mm-thick cement layer (polymethyl methacrylate) between the cut surface of the tibia and the base of the tibial component.

### Development of the finite element analysis model

Solid tetrahedral quadratic elements with mesh size less than 4 mm were used.

Separate meshes of the cortical bone, trabecular bone, polyethylene insert, tibial prosthesis tray and the cement layer were generated (Fig. 1). The corresponding material properties were simulated according to literature [17]. Young’s modulus of the polyethylene insert, titanium alloy tibial tray and the cement layer were assumed to be 0.65 GPa, 110.6 GPa and 2.65 GPa with a Poisson’s ratio of 0.46, 0.33 and 0.46, respectively. Young’s modulus of the cortical bone and trabecular bone were assumed to be 0.83 GPa and 13.4 GPa with a Poisson’s ratio of 0.3 [17]. A load of 1000 N was applied to the polyethylene insert surface along the tibia shaft axis simulating walking activity in patients with 68-kg body weight. The model was set up as a quasi-static dynamic implicit model.

### Assessment

**Fig. 4:**
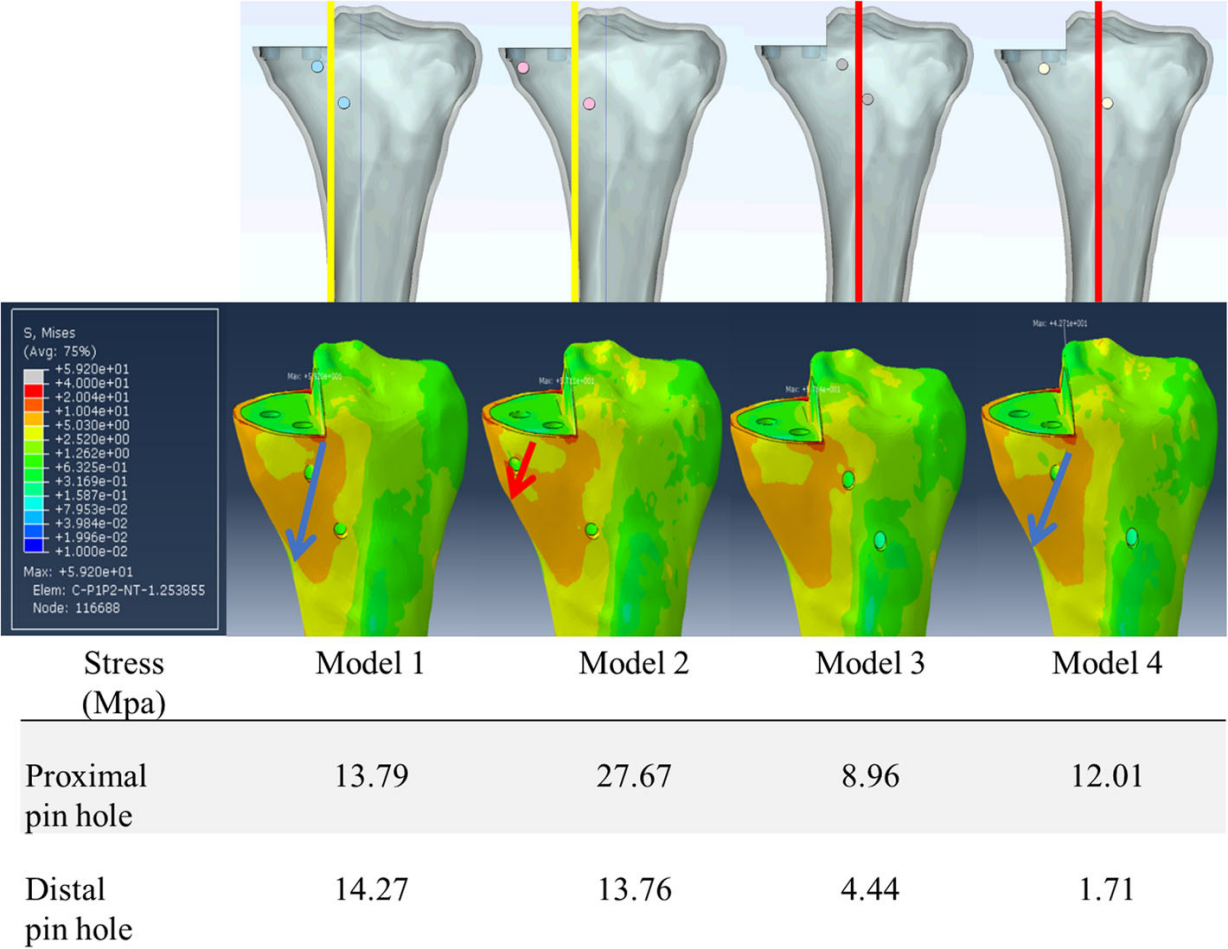
Maximum von Mises stresses on the tibial metaphyseal region in each model

To determine the effect of the resector holding pin position, the von Mises stress on the FEA model was calculated and shown on the surface of a 3-dimensional model. The stress concentration was observed in all models around the corner of the sagittal cut, pin holes and proximal tibial metaphysis to predict fracture risk. The maximum von Mises stresses on the tibial metaphyseal region in each model were also measured as they represent the general risk of fracture of the tibia (Fig. 4).

## Results

In all models, the highest stresses were observed on the sagittal cut edge region. In model 2, the high stress area around the sagittal cut, pin holes and proximal tibial metaphysis were connected. Moreover, among all four models, model 2 proximal pinhole has the highest maximum von Mises stress. For other models, the high stress areas around the pin holes were located relatively more independently without connections (Fig. 4). Besides, lower maximum von Mises stress of the pin holes were observed in model 3 (Fig. 4). Among the four models, there is a trend of increasing von Mises stress with the pin holes placed over more peripheral regions. On the contrary, more centrally placed pin holes are associated with lower von Mises stress.

## Discussion

The goal of UKA is to relieve pain and restore knee function by replacing the diseased compartment of the knee joint with an artificial implant, and the most common type is medial UKA due to high prevalence of medial osteoarthritis [21, 22]. Following UKA, immediate weight bearing walking is allowed to facilitate early rehabilitation. Therefore, structural stability of the implant and the surrounding bone are essential for patients’ early rehabilitation. The present study evaluated the difference in fracture risks due to different combination of pinholes created during tibial cutting guide placement by finite element analyses. Our result suggests that placing holding pins closer to the midline of the tibia width would result in less stress on the proximal tibia as compared to placing it closer to the tibial medial cortex or under the sagittal tibial cutting plane.

The reported incidence of periprosthetic fracture over the tibia plateau varies from zero to 10% in the literature [23, 24]. Periprosthetic fracture over the medial tibial plateau underneath the tibial baseplate is a devastating complication after UKA. Early nondisplaced stress fractures can be treated conservatively with protected weight bearing or screw fixation. However, displaced fracture with prosthesis loosening would require revision to total knee arthroplasty, of which augments and stemmed components may be needed to bridge over the fracture site [4]. Additional medial buttress plate is recommended for acute fracture fixation [4]. Protected weight bearing walking is recommended after operation. This means salvaging by a surgery of increased bone resection, delayed rehabilitation with extensive tissue dissection, and defeating the original purpose of UKA as a bone preserving, immediate weight-bearing and less-invasive surgery.

Previous reports using FEA have focused on the risks of fracture related to the coronal alignment of the tibial baseplate. It has been suggested that any valgus or varus more than 6° positioning of tibial component would increase the stress over proximal medial tibia. In order to avoid confounding factors in our finite element analysis model due to valgus/varus malalignment, we simulate the horizontal bone cut perpendicular to the mechanical axis of the tibia to achieve a neutral bone cut.

**Fig. 5:**
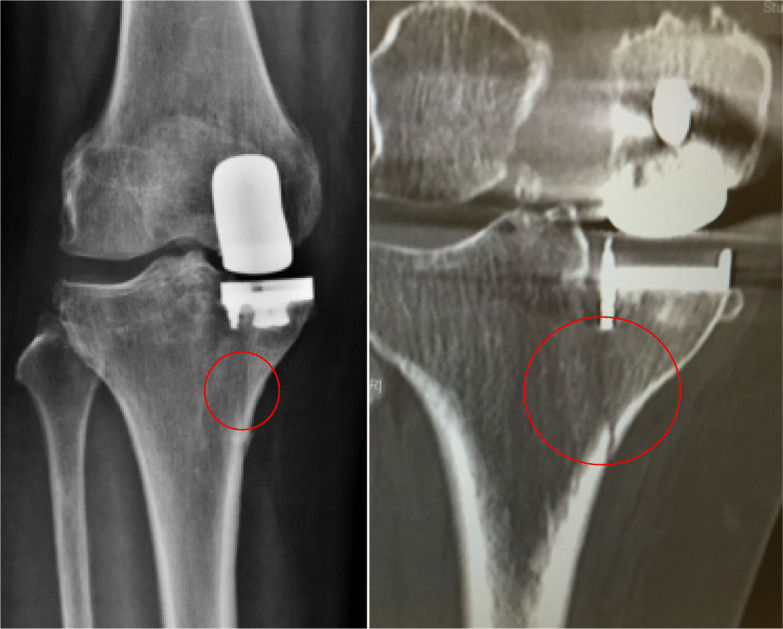
Periprosthetic fracture of UKA in a 57-year-old lady

We reported a case of unicompartmental knee arthroplasty periprosthetic fracture which we attributed to osteoporosis previously [7]. Subsequently, we encountered two more similar periprosthetic fracture cases that were with no osteoporosis or coronal malalignment and prompted us to investigate the relationship of holding pin hole position and the risk of fracture (Fig. 5, showing periprosthetic fracture of UKA in a 57-year-old lady with good past health).

In the current study, we found there are similar distinctive patterns of high stress area on all the von Mises stress graphs (Fig. 4). The first high-stress area runs along the proximal tibial medial cortex, the peripheral belt region, and the second runs underneath the sagittal tibial cutting plane, the central belt region (Fig. 4). This finding is compatible with our clinical cases in which our first one had fracture towards the proximal tibial medial cortex (peripheral belt) [7] and the second one had a fracture running directly underneath and along the sagittal tibial cutting plane (central belt) (Fig. 4). We further found that placing the tibial cutting guide holding pin inside the central and peripheral belts, as in the model 2 proximal pin, would result in excessive high stress at that point. The least stress was noted in the model 4 distal pinhole, which is far away from both central and peripheral belts; however, the model 4 proximal pinhole is also inside the central belt and so it is also associated with high stress value (Fig. 4). In model 3, both proximal and distal pinholes are away from the central and peripheral belts and the stress values are the least, suggesting this is the safest pin configuration.

Deriving from these results, each pinhole is a point of stress riser that they together create a “road” for fracture. The further this “road” of stress risers is from the central and peripheral belts, the lower is the risk of fracture. And in fact, the lowest fracture risk method would be using only one holding pin placing at the midline (as in model 4 distal pin) and together with the ankle clamp to stabilize the tibial cutting guide in a tripod fashion.

Based on this result, we have changed our clinical practice to place two holding pins along the mechanical axis of proximal tibia as in model 3, and in short term, there are no more cases of tibial fracture, although long-term results are pending. Using one pin only along the mechanical axis would reduce the risk to the minimum and is recommended to be used in osteoporotic bone. However, one pin would be less effective in counteracting the vibration during bone sawing than two pins, which may cause dislodgment of the guide, and more meticulous surgical technique is required. In the future, further studies can be conducted to understand the degree of osteoporosis that can be tolerated to safely perform UKA, for example in a single pin hole configuration, and possibly, these FEA studies may benefit from higher definition imaging like high-resolution peripheral quantitative computed tomography (HR-pQCT) [25].

Some limitations of the present study were noted. First, this is a preliminary finite element study to evaluate the risk of fracture in association to different pinholes location on the proximal tibia. The reconstructed model is based on a normal Chinese tibia female model, and generalizability to other races or gender is unknown.

Second, we simulated the virtual tibial bone cut based on the design and instruction on the surgical technique of the ZUK, a fixed bearing UKA. The FEA may potentially be variable if other brands of fixed bearing UKA or mobile bearing UKA are tested. Nevertheless, we look into other reports of UKA periprosthetic fractures and the associated brands’ design and notice they also have fractures towards the central and/or peripheral belts and the UKA brands employ two or more holding pins in their tibial cutting guides [1, 4–6, 8, 9, 23], as in ZUK. This suggests what we illustrate in this study may be also applicable to other brands of UKA.

Third, the stress values represented in the present study were applied only for comparison and risk evaluation among the four models. The correlation between stress values and actual practical failure may need further validation by mechanical testing like using cadaveric study, though they appear to correspond to our clinical cases.

Finally, only partial parameters of the design and human structures were extracted for modelling in the present study. For example, integrity of surrounding soft tissue envelope may alter the stress distribution, and so the risk in actual clinical practice may still be variable.

## Conclusion

The present study evaluated the stress distribution in adopting different positions of the tibial cutting guide holding pins and subsequent risks of periprosthetic fracture after UKA. It confirmed our hypothesis that certain tibial cutting guide pin configuration posted increased risk for tibial periprosthetic fracture. Stress concentration was observed along the sagittal cutting plane and around pin holes. A configuration of these in close proximity would cause connection of stress riser and hence development of fracture. This study has provided a clear evidence in against using such pin configuration.

Two holding pins along mechanical axis is the pin configuration with the lowest risk of fracture when a standard two holding pins method is employed. In osteoporotic bone, only one pin along the mechanical axis for holding tibial cutting guide may be considered to further reduce fracture risk.

## Abbreviations

UKA: Unicompartmental knee arthroplasty
TKA: Total knee arthroplasty
BMD: Bone mineral density
FEA: Finite element analysis
